# 

**Published:** 1904-06-11

**Authors:** 


### NOTICE TO CORRESPONDENTS.

All MSS., Letters, Books for Review, and other matters intended for the Editor should be addressed The Editor, "The Hospital," The Hospital Buildings, 28 & 29 Southampton Street, Strand, W.C.

The Editor cannot undertake to return rejected MS., even when accompanied by stamped directed envelope.

**Notice to Advertisers and Subscribers.**

All Advertisements, Orders for Copies of The Hospital, and Business Communications should be addressed to **THE MANAGER (Not to the Editor),** The Hospital, 28 & 29 Southampton Street, Strand, London, W.C.

The Cost of Subscription, post free, is as follows:—Per week, 3½d.; per quarter, 3s. 3d.; per half-year, 6s. 6d.; per annum, 13s. Foreign and Colonial:—Per annum, 19s. 6d., payable, in all cases, in advance.

**NOTICE.—Vol. XXXV. of "The Hospital" (October 1903 to March 1904), in handsome cloth binding, is now on sale at the office of this Journal, price 10s. 6d. Cases for binding the Numbers contained in this Volume 2s. Index to Vol. XXXV., 3½d., post free.**

**The Monthly part for June is now ready. Price 1s., by post, 1s. 3d.**

THE Chelsea Hospital for Women has received a donation of £200 to provide for increase of work, from its benefactress, H. M. E., and £10 10s. from the Skinners' Company.

### MEDICAL APPOINTMENTS.

**John Bowes**, M.D.Edin., C.M., Medical Officer and Public Vaccinator for the Failsworth District of the Prestwich Union. **Herbert W. Carson**, F.R.C.S.Eng., Surgeon to the Tottenham Hospital. **John Costelloe**, M.B., Ch.M.Melb., Government Medical Officer and Vaccinator at Babranald, N.S.W. **L. Erasmus Ellis**, M.D.Brux., M.R.C S.Eng., L.R.C.P.Lond., L.S.A.Lond., Clinical Assistant to St. John's Hospital for Diseases of the Skin, Leicester Square, W.C. **E. Florance**, M.D., Visiting Surgeon to the Gaol at Cootamundra, N.S.W. **Geo. E. Froggatt**, M.B., B.S.Durh., Medical Superintendent of the Shoreditch Workhouse and Infirmary. **A. Leonard Fuller**, M.R.C.S.Eng., L.R.C.P. Lond., Honorary Assistant Surgeon to the Royal United Hospital, Bath. **O. T. Williams**, M.R.C.S., F.R.C.P.Lond., Resident Medical Officer, West Derby Union Infirmary.

HOUSE APPOINTMENTS AT ST. THOMAS'S HOSPITAL.—The following gentlemen have been selected as House Officers from Tuesday, June 7th, 1904:—Resident House Physicians: **G. C. Adeney**, M.R.C.S., L.R.C.P. (*Extension*); **E. A. Ross**, M.B., B.C.Cantab. (*Extension*); **H. C. Lecky**, M.A., M.B., B.Ch.Oxon.; **C. H. Latham**, M.R.C.S., L.R.C.P. House Physicians to Out-Patients: **B. Higham**, M.R.C.S., L.R.C.P.; **W. Haward**, M.B., B.S.Durh., M.R.C.S., L.R.C.P. Resident House Surgeons: **W. L. Harnett**, M.A., M.B., B.C.Cantab., M.R.C.S., L.R.C.P. (*Extension*); **H. I. Pinches**, M.A., M.B., B.C.Cantab., M.R.C S., L.R.C.P. (*Extension*); **T. Guthrie**, M.A., M.B., B.C.Cantab., M.R.C.S., L.R.C.P. (*Extension*); **L. E. Wigram**, M.A., M.B., B.C.Cantab. (*Extension*). House Surgeons to Out-patients: **H. S. Bennett**, M.R.C.S., L.R.C.P. (*Extension*); **N. C. Carver**, B.A., B.C.Cantab., M.R.C.S., L.R.C.P. (*Extension*); **A. C. Birt**, M.R.C.S., L.R.C.P. (*Extension*); **G. T. Birks**, M.A., M.B., B.C.Cantab. (*Extension*). Obstetric House Physicians: (*Senior*) **C. N. Sears**, M.B., B.S.Lond., M.R.C.S., L.R.C.P.; (*Junior*) **J. P. Hedley**, M.A., M.B., B.C.Cantab., M.R.C.S., L.R.C.P. Special Departments: (Throat) **W. Ibbotson**, M.R.C.S., L.R.C.P. (*Extension*); **G. R. Rickett**, M.A., B.C.Cantab.; (Skin) **G. R. Rickett**, M.A., B.C.Cantab.; (Ear) **W. Ibbotson**, M.R.C.S., L.R.C.P. (*Extension*).

**"The Hospital" Institutional Library.**

"Hospitals and Asylums of the World." 4 Vols., with a Portfolio of Plans, complete £12 12s.

"Burdett's Hospitals and Charities, 1904." 5s. net.

"Burdett's Official Nursing Directory." 8s. net.

"Cottage Hospitals, General, Fever, and Convalescent." 10s. 6d.

"Uniform System of Accounts for Hospitals and Public Institutions." New Edition. 4s. net.

"Hospital Expenditure: The Commissariat." 2s. 6d.

"A Legal Handbook for the Use of Hospital Authorities." 2s. 6d.

"The Cottage Hospital Case Book or Register of Patients." 10s. and 12s. 6d.

All these are published by the SCIENTIFIC PRESS, Ltd., and may be obtained through any bookseller, or direct from the publishers, 28 and 29 Southampton Street, Strand, London, W.C.

### Medical Appointments.

### The Midwives Act.

### IMPORTANT.

The above Act is destined to bring about many changes in the practice of Midwifery by Midwives. In the past a woman however incompetent or unfit could call herself a midwife; under the Midwives Act it will be illegal for a woman to call herself a midwife unless she has fulfilled certain legal requirements. The legal requirements of the Act differ according as the woman is or is not already a Midwife or holds or does not hold a certificate in Midwifery. In

### HOW TO BECOME A CERTIFIED MIDWIFE,

**By Dr. E. L. C. APPEL, M.B., B.S., B.Sc.Lond.,**

these legal requirements are all set forth in such a way that a woman can by referring to the table of contents readily see which of them are applicable in her case. The book also contains a list of Midwifery Training Schools recognised by the Central Midwives Board which should prove of much value to anyone desirous of training in this important branch of the Nursing Profession.

The price of the book is 1/- net, 1/1 post free, and it can **now** be obtained of all Booksellers, or direct from the Publishers at the address given below.

---

*JUST PUBLISHED.*

### A COMPLETE HANDBOOK OF MIDWIFERY.

**By J. K. WATSON, M.D. (Edin.).**

This handbook has been designed to meet the requirements of the Central Midwives Board, and is an up-to-date text-book of **all** that the midwife requires to know of the subject treated. The work is produced with the object of rendering it unnecessary for midwives, during their preparation for examination, to consult the larger and more expensive works on the subject written especially for medical men.

**The illustrations are very complete, numbering some 143 separate diagrams, most of which have been specially prepared for this book.**

Crown 8vo. about 350 pp., bound in cloth boards, 6/- net, 6/4 post free.

### THE MIDWIVES' POCKET BOOK.

**By HONNOR MORTEN.**

**With Appendix containing particulars and Rules of the Central Midwives Board.**

Small crown 8vo. in cloth boards, 1/- post free.

---

**Complete Catalogue of Nursing Manuals post free on application.**

*Of all Booksellers or of*

